# Menopausal status, age at natural menopause and risk of diabetes in China: a 10-year prospective study of 300,000 women

**DOI:** 10.1186/s12986-022-00643-x

**Published:** 2022-02-05

**Authors:** Meng Wang, Wei Gan, Christiana Kartsonaki, Yu Guo, Jun Lv, Zhengming Chen, Liming Li, Ling Yang, Min Yu

**Affiliations:** 1 Department of NCDs Control and Prevention, Zhejiang Provincial Center for Disease Control and Prevention, 3399 Binsheng Road, Hangzhou, 310051 China; 2 Clinical Trial Service Unit and Epidemiological Studies Unit (CTSU), Medical Research Council Population Health Research Unit, Nuffield Department of Population Health, Big Data Institute, University of Oxford, Old Road Campus, Roosevelt Drive, Oxfordshire, Oxford, OX3 7FZ UK; 3 Chinese Academy of Medical Sciences, Dong Cheng District, Beijing, 100864 China; 4 Department of Epidemiology and Biostatistics, School of Public Health, Peking University Health Science Center, 38 Xueyuan Road, Haidian District, Beijing, 100191 China

**Keywords:** Diabetes, China, Women, Menopause

## Abstract

**Background:**

Menopause characteristics have been implicated in future health consequences, yet little is known about its relevance to diabetes. We examined the associations of menopausal status and age at natural menopause with diabetes risk in Chinese women.

**Methods:**

We used prospective data of the China Kadoorie Biobank study that recruited 302,522 women aged 30–79 years in 2004–2008 from 10 areas across China. During average 10.8 years of follow-up, 11,459 incident diabetes cases were recorded among 281,319 women without prior diabetes diagnosis at baseline. Cox proportional hazards models were used to estimate adjusted hazard ratios (HRs) with 95% confidence intervals (CIs) for incident diabetes associated with menopausal status and age at natural menopause.

**Results:**

Overall, the mean (SD) age at natural menopause was 48.2 (4.4) years among 141,789 post-menopausal women. Naturally peri-, or post-menopausal women were at higher risk of diabetes, with HRs of 1.17 (95% CI 1.06–1.29) and 1.15 (1.06–1.25) compared with pre-menopausal women, adjusting for several potential confounders. Among women who had natural menopause, the HR of diabetes was 1.14 (1.01–1.30), 1.01 (0.93–1.09), 1.10 (1.04–1.16), and 1.10 (1.01–1.20) for menopause at ages less than 40, 40–44, 50–53, and 54 years or older, respectively, relative to 45–49 years.

**Conclusions:**

In this study, we found that women with naturally peri-, or post-menopausal status had higher risk of developing diabetes. Besides, among the post-menopausal women, both earlier and later age at natural menopause were associated with increased risk of diabetes.

**Supplementary Information:**

The online version contains supplementary material available at 10.1186/s12986-022-00643-x.

## Background

Diabetes is one of the largest public health challenges of the twenty-first century. Estimated by the International Diabetes Federation (IDF), a staggering 537 million adults were living with diabetes in 2021 worldwide and this figure was expected to increase to 643 million by 2030 [1]. The past decades has witnessed substantial growth of diabetes in China. During 1980 and 2017, the prevalence of diabetes among Chinese adults increased from 0.67 to 11.2%, resulting in 129.8 million patients [2, 3]. Meanwhile, within Chinese populations, epidemiological evidence further indicated higher diabetes prevalence and related mortality among women than men in their 60 s or older [4–6], which highlighted the necessity to better investigate the extra risk factors in these susceptible groups.

Menopause is a point in time 12 months after a woman's final menstrual period, marking the permanent cessation of ovarian function. As ovaries are considered the main source of female hormones, great public health concerns have been raised on the health consequences of menopause. In literature, the post-menopausal status coincided with increased risks for multiple chronic conditions among women [7], and moreover, findings suggested that the earlier and later age at menopause was associated with higher risk of cardiovascular diseases [8] and cancers (e.g., breast and endometrium cancer) [9, 10], respectively.

Diabetes is an important risk factor for several cardiovascular diseases and cancers. Several studies on the association between menopause and diabetes have been reported, but results are inconsistent. Some findings showed that post-menopausal women were more likely to have diabetes than pre-menopausal women [11–13], while others did not [14, 15]. The mixed associations with diabetes also emerged as regards the age at menopause. In both cross-sectional [16] and longitudinal studies [17, 18], earlier menopause was found to be associated with increased odds of diabetes. Recent cohort studies also reinforced the inverse association with observations that later menopause was associated with lower risk of diabetes [19, 20]. However, in contrast, some authors reported that women who had later menopause experience were more likely to have diabetes [12, 21].

Therefore, based on data from a prospective cohort study of the China Kadoorie Biobank (CKB), we aimed to examine the associations of menopausal status and age at natural menopause with the risk of diabetes in Chinese women.

## Methods

### Study design and population

Details on the CKB study design and population have previously been described elsewhere [22]. Briefly, the baseline survey was conducted from 2004 to 2008 in 10 diverse regions across China, with 512,715 Chinese adults (302,522 women) aged 30–79 were successfully recruited. Data about sociodemographic characteristics, smoking, alcohol drinking, diet, physical activity, general health (e.g., disease history and current medication use), and family history of disease (e.g., diabetes and cancers) were collected using an interviewer administered laptop-based questionnaire. Separately, women were asked about the reproductive history (e.g., age at menarche, parity, age at birth, breastfeeding duration for each live birth, and menopausal status and age at menopause), relevant surgery treatment, and oral contraceptive (OC) use. Anthropometric measurements, such as height and weight, and physical measurements, such as blood pressure and random plasma glucose (RPG) were took by health workers, using calibrated instruments according to standardized protocols. RPG levels were measured on-site using the Johnson and Johnson SureStep Plus System (LifeScan, Milipitas, California, USA). Participants without self-reported diabetes with a RPG level of 7.8–11.0 mmol/L were invited to undergo fasting glucose testing the next day. Participants who reported a history of physician-diagnosed diabetes were considered to be self-reported diabetes. Screen-detected diabetes was defined as not having self-reported diabetes but having a RPG level ≥ 7.0 mmol/L with more than 8 h since last food, RPG level > 11.1 mmol/L with less than 8 h since last food, or fasting plasma glucose level ≥ 7.0 mmol/L on subsequent testing [23].

### Assessment of menopausal status and age at natural menopause

Women were asked to answer the question “Have you had your menopause?” at baseline, with response options and relevant explanations as follows: (1) no (having regular menstrual cycle); (2) yes, currently (having irregular menstrual cycle but before 12 months of amenorrhea); (3) yes, had menopause (having amenorrhea for 12 months or more). The menopausal status of women was identified as pre-, peri- or post-menopausal, when they reported that they had not, were currently, or had menopause, respectively. Furthermore, among women who had menopause, the age of completion of menopause was then asked. In this analysis, the age at natural menopause was grouped as age less than 40 (i.e., premature menopause), 40–44 (i.e., early menopause), 45–49 (as reference), 50–53, and 54 year or older (i.e., later age at menopause).

### Follow-up and endpoint definition

Participants were followed up for cause-specific morbidity and mortality, mainly through linkage with the disease monitoring systems. The vital status of each participant was obtained from the local disease surveillance points system death registries and residential records, and supplemented by active confirmation through street committee or village administrators. Information on non-fatal outcomes was collected through linkage with established disease registries and national health insurance system, which has almost universal coverage (approximately 99%) and captures episodes of new-onset diabetes for both outpatients and hospitalized patients. Fatal and nonfatal events were coded according to the International Classification of Diseases, 10th Revision (ICD-10), and blinded to baseline information. The primary outcome of the present analysis was incident diabetes (E10–E14). The person-years at risk were calculated from the baseline to diabetes diagnosis, death, loss to follow-up, or the study termination (December 31, 2017), whichever occurred first.

### Statistical analysis

Among 302,522 women recruited in the baseline survey, we excluded those with missing data on menopausal status (n = 47), a history of surgical menopause (n = 1,240), cancer (n = 1,408), or self-reported diabetes or screen-detected diabetes (n = 18,508). After these exclusions, 281,319 women were included in the present analysis.

Multivariable Cox proportional hazards models were used to estimate the hazard ratios (HRs) with 95% confidence intervals (CIs) for the associations between menopausal status, age at natural menopause and risk of diabetes. The analysis for menopausal status was conducted among all included women, while for age at natural menopause was confined to post-menopausal women only. These analyses used the time in study as the underlying time scale and stratified by age at risk (5-year intervals) and study region. The proportional hazards assumption for the Cox model was checked using Schoenfeld residuals, and no violation was found.

Adjustments for confounding factors were conducted in four sequential models. In model 1, only education (no formal school, primary school, middle school, high school, college/university) and household income (< 10 k, 10–20 k [10,000–19,999], 20–35 k [20,000–34,999], ≥ 35 k yuan) were adjusted. Model 2 was further adjusted for health behaviors of smoking (never, occasional, current regular), alcohol drinking (never, occasional, current regular), physical activity (Metabolic Equivalents of Task, h/d), and anthropometric measurements including body mass index (BMI, kg/m^2^, underweight [< 18.5], normal weight [18.5–23.9], overweight [24.0–27.9], obesity [≥ 28.0]) [24] and waist circumference (< 80 and ≥ 80 cm) at baseline. Model 3 was adjusted for all variables in model 2 plus health status of hypertension, and family history of diabetes. Model 4 was additionally adjusted for other reproductive factors of age at menarche, number of live births, age at first birth, breastfeeding duration per child, and OC use, which we took as our primary analysis. On the basis of model 4, associations between age at natural menopause and risk of diabetes were compared within subgroups of post-menopausal women defined by region, birth cohort, education, smoking, alcohol drinking, BMI, hypertension, and other reproductive factors including age at menarche, OC use, number of live birth, age at first birth and duration of breastfeeding per child. To evaluate the robustness of our estimates, sensitivity analyses were conducted with excluding those (1) who smoked, drank alcohol, or used OC; and (2) who were aged < 57 years at baseline to avoid any potential distortion of the distribution of age at natural menopause among the younger age group. All analyses were performed using SAS version 9.4 (SAS Institute, Inc., Cary, NC) and R version 4.1.1 (The R Foundation for Statistical Computing). All statistical tests were based on the two-sided 5% level of significance.

## Results

### Characteristics of study participants

Among the 281,319 women included, the mean (SD) age at baseline was 50.9 (10.4) years. 43.5% of the women were urban residents and 25.0% had no formal education. Few women were current regular smokers (2.3%) or alcohol drinkers (3.6%), and 9.5% women had ever used OC. At baseline, 44.6% of the women were pre-menopausal, 5.0% peri-menopausal, and 50.4% post-menopausal. Compared with naturally pre-menopausal women, peri- or post-menopausal women tended to be urban residents, less educated and physically active, with higher waist circumference and higher proportion of smoking, overweight/obesity, OC use, and to have higher age at menarche, more children and longer breastfeeding duration. Among the 141,789 post-menopausal women, the mean (SD) age at natural menopause was 48.2 (4.4) years. Compared with women who had later menopause, those having earlier menopause were, on average, younger and leaner at baseline, more likely to be rural residents, smoke more, and be more active, and to have lower age at menarche, with a higher proportion of nulliparity and lack of breastfeeding (Table 1).

**Table 1:** Baseline characteristics of CKB women according to the menopausal status and age at natural menopause

Characteristics	Overall	Menopausal status			Age at menopause				
		Pre-	Peri-	Post-	< 40	40–44	45–49	50–53	≧ 54
No. of women	281,319	125,494	14,036	141,789	5,890	16,523	58,998	49,346	11,032
Mean age at baseline, year	50.9	41.9	49.7	59.1	56.0	58.1	58.7	59.5	62.3
Birth cohorts, %									
1920s–1930s	9.5	0.03	0.06	18.7	22.0	21.1	18.2	17.1	23.2
1940s	19.5	0.1	1.2	38.4	25.6	31.9	35.3	40.7	61.3
1950s–1970s	71.0	99.9	98.8	42.9	52.4	47.0	46.5	42.2	15.5
Urban resident, %	43.5	41.0	48.6	45.2	41.7	38.4	43.2	49.2	50.0
No formal school, %	25.0	11.7	25.4	36.7	37.9	38.1	37.8	34.3	38.1
Lifestyle factors and anthropometric measurements, % or mean									
Current regular smoker	2.3	0.9	1.3	3.6	4.0	4.4	3.8	3.2	3.3
Current regular drinker	3.6	3.6	4.0	3.5	4.0	3.8	3.5	3.5	3.4
Body mass index, kg/m^2^									
Overweight (24.0–27.9)	33.0	31.5	38.1	33.9	32.6	32.1	33.0	35.1	36.8
Obesity (≧ 28.0)	11.1	9.2	13.2	12.6	11.9	11.0	11.9	13.4	15.7
Waist circumference, cm	78.7	77.0	79.4	80.1	79.4	79.3	79.7	80.5	81.5
Physical activity, MET, h/d	20.8	24.8	21.8	17.2	18.8	18.0	17.5	16.7	15.8
Reproductive factors, % or mean									
Age at menarche, year	15.4	14.9	15.4	15.9	15.7	15.7	15.9	16.0	16.3
Nulliparous	1.3	1.5	0.9	1.3	4.8	1.5	1.2	0.9	0.8
Oral contraceptive pill used	9.5	8.7	13.9	9.8	7.3	8.5	9.9	10.6	9.7
No. of live births^†^	2.2	1.6	1.7	2.8	2.6	2.9	2.8	2.7	3.2
Age at first birth, year^†^	23.4	23.7	24.3	23.0	22.7	22.8	23.0	23.2	22.7
Never breastfed^†^	2.8	3.6	3.2	2.2	3.5	2.6	2.1	2.0	1.7
Breastfeeding per child, month^†^	14.3	13.4	14.2	15.0	14.8	15.1	15.2	14.7	15.2

*CKB* China Kadoorie Biobank, *MET* metabolic equivalents of task

^†^Among parous women only

### Associations between menopausal status, age at natural menopause and diabetes risk

**Fig. 1:**
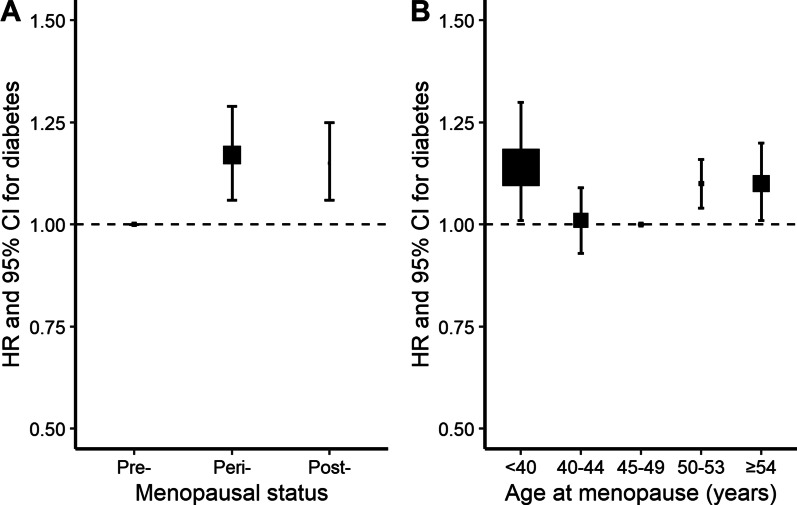
Associations of menopausal status and age at natural menopause with diabetes risk. Specifically, **A** for menopausal status; **B** for age at natural menopause among post-menopausal women only. Squares represent the adjusted hazard ratios (HRs) compared with the reference group of pre-menopausal status (**A**) and menopause at age 45–49 years (**B**), respectively, with area inversely proportional to the number of cases. Vertical lines indicate the corresponding 95% confidence intervals (CIs)

**Fig. 2:**
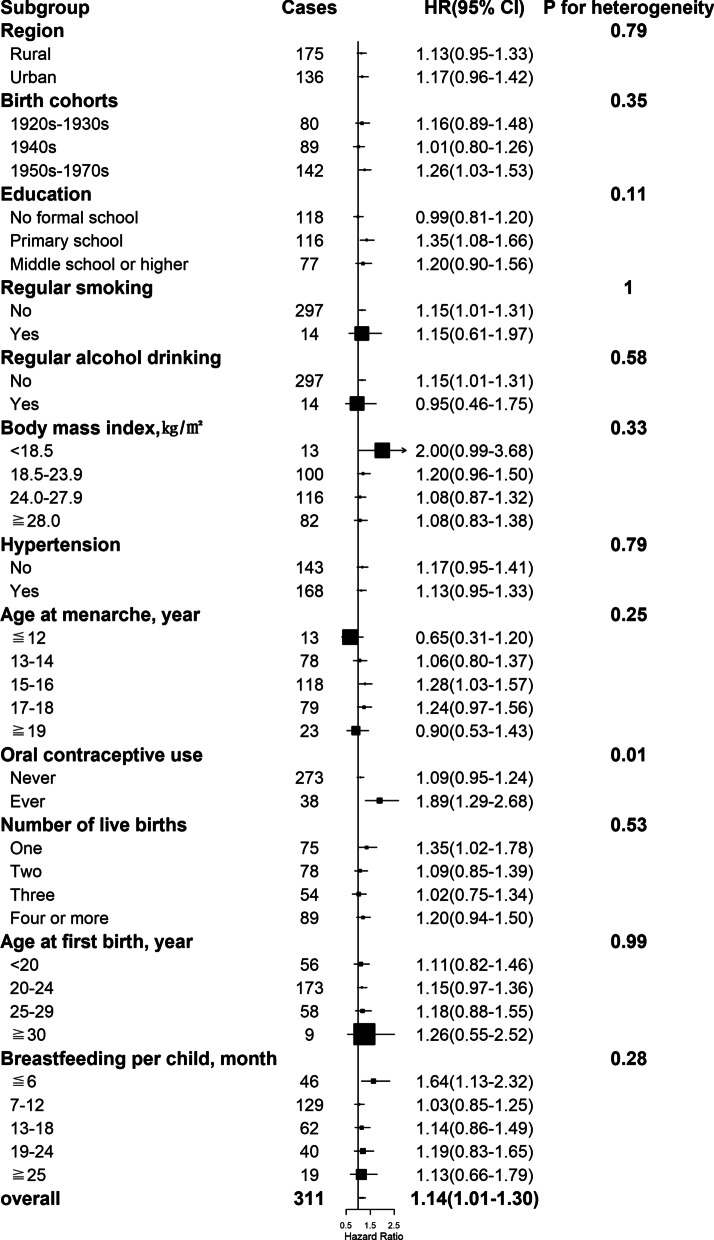
Associations with natural menopause at age < 40 years (i.e., premature menopause) and diabetes risk within subgroups. Squares represent the adjusted hazard ratios (HRs) compared with the reference group of menopause at age 45–49 years, with area inversely proportional to the number of cases. Horizontal lines indicate the corresponding 95% confidence intervals (CIs)

**Fig. 3:**
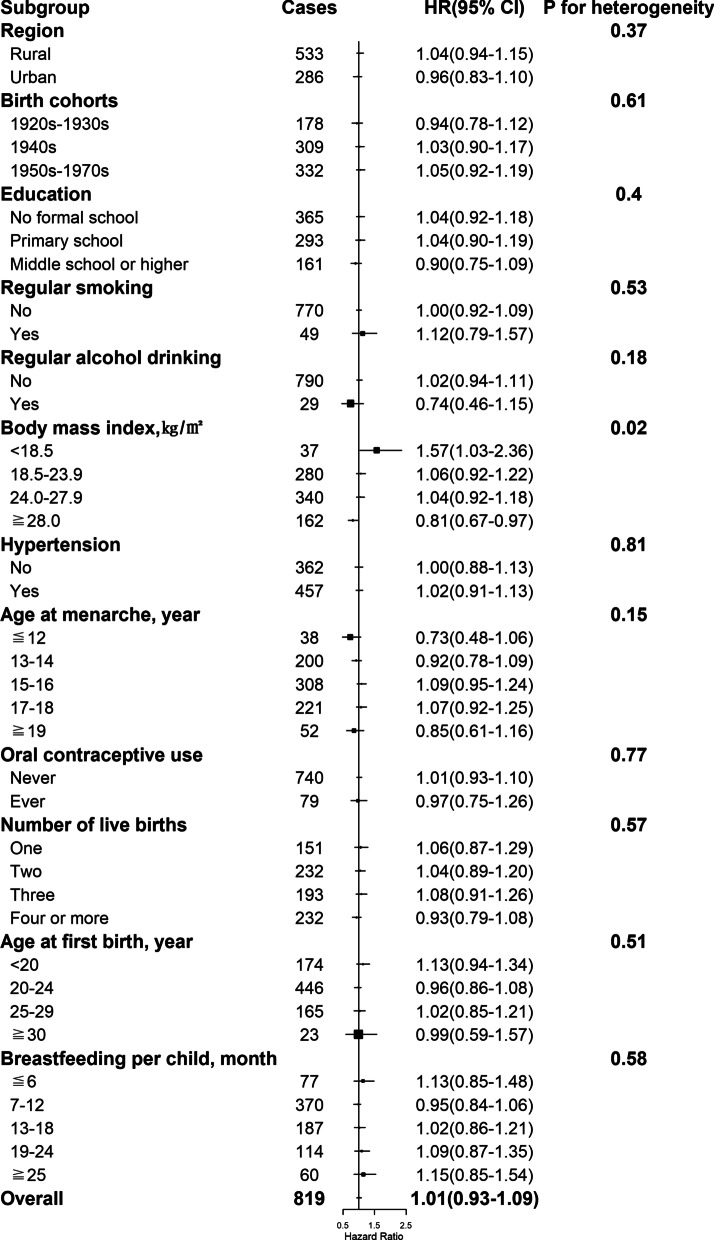
Associations with natural menopause at age 40–44 years (i.e., early menopause) and diabetes risk within subgroups. Squares represent the adjusted hazard ratios (HRs) compared with the reference group of menopause at age 45–49 years, with area inversely proportional to the number of cases. Horizontal lines indicate the corresponding 95% confidence intervals (CIs)

**Fig. 4:**
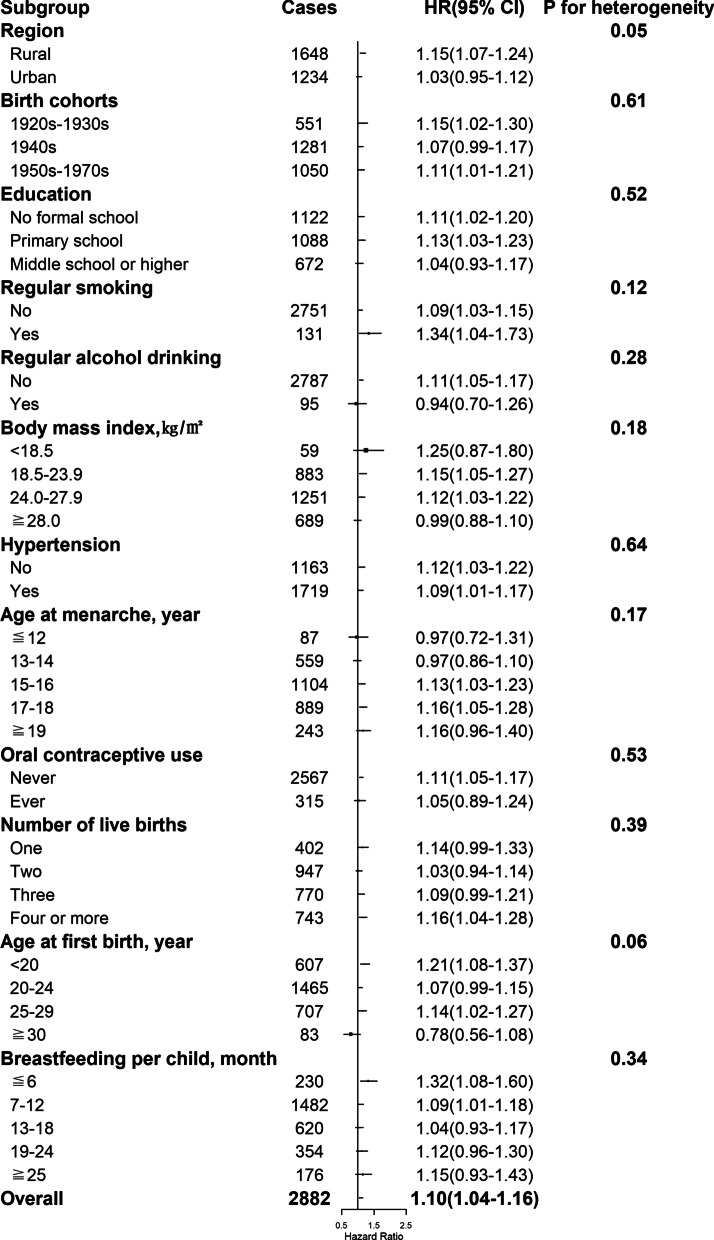
Associations with natural menopause at age 50–53 years and diabetes risk within subgroups. Squares represent the adjusted hazard ratios (HRs) compared with the reference group of menopause at age 45–49 years, with area inversely proportional to the number of cases. Horizontal lines indicate the corresponding 95% confidence intervals (CIs)

**Fig. 5:**
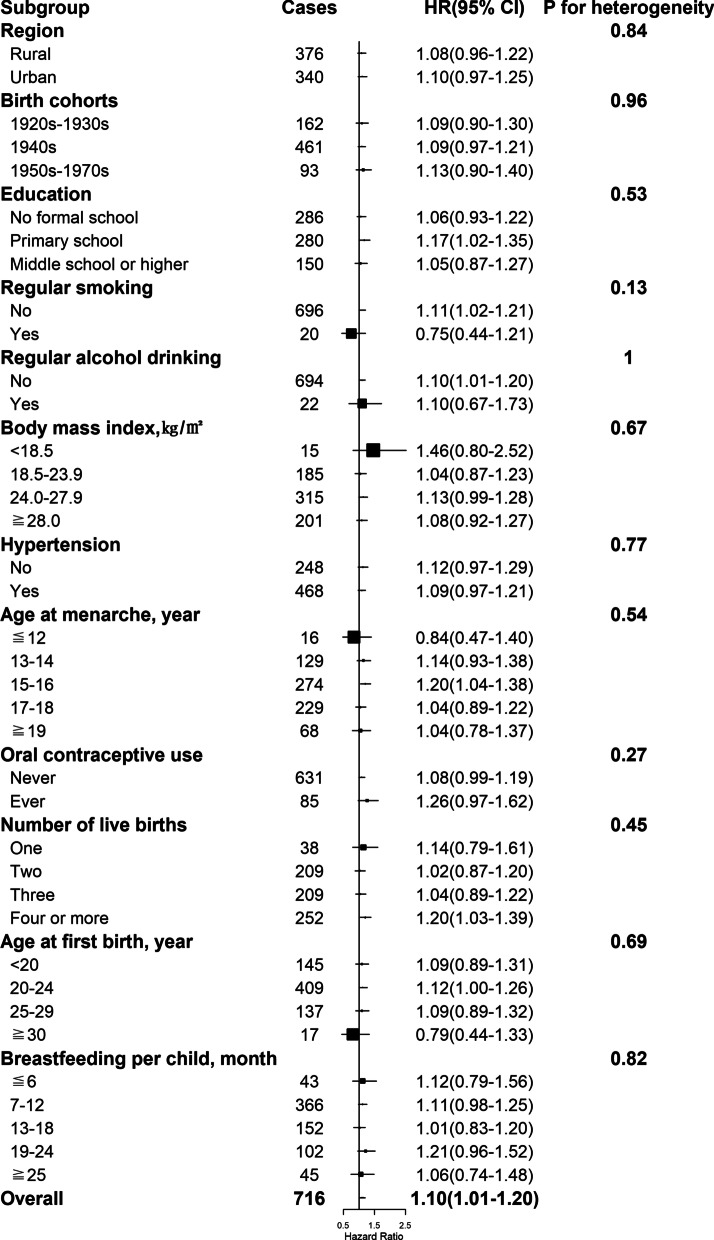
Associations with natural menopause at age ≧54 years (i.e., later age at menopause) and diabetes risk within subgroups. Squares represent the adjusted hazard ratios (HRs) compared with the reference group of menopause at age 45–49 years, with area inversely proportional to the number of cases. Horizontal lines indicate the corresponding 95% confidence intervals (CIs)

By the end of 2017, a total of 11,459 women developed diabetes during 3,050,083 person-years of follow-up (mean follow-up duration of 10.8 years). After adjustment for potential confounders, including sociodemographic characteristics, health behaviors, and other reproductive factors, compared with naturally pre-menopausal women, peri-, or post-menopausal women had statistically significantly higher risk of diabetes, with the HRs of 1.17 (95% CI 1.06–1.29) and 1.15 (1.06–1.25) (Table 2 and Fig. 1A). Among post-menopausal women, the HR of incident diabetes was 1.14 (1.01–1.30), 1.01 (0.93–1.09), 1.10 (1.04–1.16), and 1.10 (1.01–1.20) for natural menopause at ages less than 40, 40–44, 50–53, and 54 years or older, respectively, relative to 45–49 years (Table 2 and Fig. 1B). No heterogeneity was observed for the associations of age at natural menopause with incident diabetes by region, birth cohort, education, smoking, alcohol drinking, hypertension, and other reproductive factors including age at menarche, number of live birth, age at first birth and duration of breastfeeding per child (all *P* for heterogeneity ≥ 0.05) (Figs. 2, 3, 4, 5 and Additional file 1: Table S1), although the associations of incident diabetes with premature menopause and early menopause differed statistically as regards OC use (*P* for heterogeneity = 0.01) and BMI (*P* for heterogeneity = 0.02), respectively (Figs. 2, 3 and Additional file 1: Table S1). In the sensitivity analyses, although the HRs varied slightly, the associations of menopausal status and age at natural menopause with diabetes risk were broadly consistent among the subsets of women who never smoked, drank alcohol, or used OC, and who were aged ≥ 57 years at baseline (Additional file 1: Table S2).

**Table 2:** Adjusted hazard ratios (95% CIs) of diabetes by the menopausal status and age at natural menopause

	Total/cases	Model 1	Model 2	Model 3	Model 4
Menopausal status					
Pre-	125,494/3,055	1.00	1.00	1.00	1.00
Peri-	14,036/664	1.18 (1.07–1.30) *	1.18 (1.07–1.30) *	1.18 (1.07–1.30) *	1.17 (1.06–1.29) *
Post-	141,789/7,740	1.11 (1.02–1.20) *	1.15 (1.06–1.25) *	1.16 (1.07–1.25) *	1.15 (1.06–1.25) *
Age at menopause, year					
< 40	5,890/311	1.15 (1.01–1.29) *	1.15 (1.01–1.29) *	1.15 (1.01–1.31) *	1.14 (1.01–1.30) *
40–44	16,523/819	1.00 (0.93–1.08)	1.01 (0.93–1.09)	1.01 (0.93–1.09)	1.01 (0.93–1.09)
45–49	58,998/3,012	1.00	1.00	1.00	1.00
50–53	49,346/2,882	1.14 (1.08–1.20) *	1.10 (1.05–1.16) *	1.11 (1.05–1.17) *	1.10 (1.04–1.16) *
≧ 54	11,032/716	1.18 (1.09–1.28) *	1.11 (1.02–1.20) *	1.10 (1.00–1.19) *	1.10 (1.01–1.20) *

Model 1 adjusted for education, household income; model 2 adjusted for model 1 plus health behaviors of smoking, alcohol drinking, physical activity (Metabolic Equivalents of Task, h/d), and anthropometric measurements including body mass index, waist circumference; model 3 adjusted for model 2 plus health status of hypertension, and family history of diabetes; model 4 adjusted for model 3 plus reproductive factors of age at menarche, number of live births, age at first birth, breastfeeding duration per child, and oral contraceptive use

All models were stratified by age and study area

^*^Significant results

## Discussion

Based on nearly 300,000 middle-aged women from 10 diverse regions in China, we found that naturally peri-, or post-menopausal women had significantly increased risk of developing diabetes compared with pre-menopausal women of the same age, after adjustment for potential confounders. Moreover, among post-menopausal women, we observed that both premature menopause and later age at natural menopause were associated with incident diabetes, and these associations were broadly consistent across most subgroups of women. To our knowledge, this is the first large prospective study in mainland China to examine the menopause characteristics with diabetes risk.

There is mechanistic evidence that the menopausal transition and post-menopause stages in women is accompanied by changes in sex steroid hormones [25, 26], body composition and body fat distribution [27], and lipid and metabolic profiles [28, 29] that are relevant to diabetes risk. However, to date it is not clear what role of specific peri-, or post-menopausal status plays in a woman’s risk of developing diabetes across population-based studies. For one thing, interests of most prior research have been focused on the health consequences of menopause, while the possible effects of peri-menopause is rarely reported. Recently, the peri-menopausal status has been implicated in the excess risks of multiple chronic conditions, such as cardiovascular diseases [30], metabolic syndrome [31], depression [32], and urinary incontinence [33] in longitudinal studies. Questions remain, however, about the relevance to diabetes risk of peri-menopause in women. The present prospective study filled the current evidence gap and showed that the peri-menopausal status was significantly associated with increased risk of diabetes in Chinese women. Assuming a causal association, the period of transition to post-menopause should be consider as a critical window for monitoring women’s health during midlife and projecting early intervention strategies to reduce diabetes risk. Meanwhile, limited by the relatively smaller proportion of peri-menopausal women (only 5.0% of the overall women) involved in this analysis, more prospective studies centered at the peri-menopause period with a greater sample size are warranted to confirm our findings. For another, epidemiological studies on the association between post-menopausal status and diabetes yielded inconclusive results with either a positive or no association. Specifically, in several large cross-sectional analyses conducted in Japan (n = 10,878), China (n = 16,114), and Italy (n = 44,694), findings consistently showed that the naturally post-menopausal women were more likely to have type 2 diabetes (odds ratio, OR [95% CI] 1.40 [1.03–1.89], 1.54 [1.10–2.14], and 1.38 [1.03–1.84], respectively) compared with pre-menopausal women, after adjustment for age [11–13]. Nevertheless, the notable increase in the odds of diabetes was not confirmed across the few longitudinal studies, with no association was observed [14, 15]. The present cohort study, with analysis results that post-menopausal women were at higher risk of diabetes (HR [95% CI] 1.15 [1.06–1.25]) after allowance for the effect of age, possibly for the first time, provided prospective evidence linking natural menopause with diabetes risk.

Moreover, in the analysis of post-menopausal women, significantly increased HRs of incident diabetes were observed for premature menopause and later age at natural menopause. Although there are large differences in the items of age range at menopause, study design, sample size and population, and potential confounders adjustment, similar associations have been seen in previous literature. In a cross-sectional analysis of 5,063 Chinese post-menopausal women from the jinchang Cohort Study, authors found that natural menopause at ages ≤ 40 and ≥ 56 years were borderline significantly or significantly associated with higher prevalence of diabetes [12]. Similarly, a recent prospective cohort analysis of data from the Women’s Health Initiative, examining 124,379 post-menopausal women aged 50–79 years, suggested that those with menopause before age 45 and after 55 years had increased risk of diabetes [21]. Regarding the potential mechanisms accounting for these reported associations between age at menopause and diabetes, prior studies have proposed that the adverse effects on insulin and glucose levels induced by both short and prolonged endogenous estrogen exposure [25, 34–38] should be considered.

The major strengths of the present study, including the prospective design, large sample size, and diversity of areas covered, contribute to the generalization of study findings to general population in China. Moreover, the completeness of data collection, stringent case ascertainment via comprehensive follow-up systems, and the wide adjustment for potential confounders simultaneously limit the possible confounding bias in the analyses. Some limitations need to be taken into account. Despite allowance for a comprehensive set of potential confounders, residual confounding from other known or unknown risk factors may still exist in this observational study. The information of menopause is relied on self-reports and may have been subject to reporting bias, resulting in misclassification of the menopausal status. Notably, although evidence has shown that recalled and actual menopausal age is reasonably well correlated [39], among the post-menopausal women, the mean age at baseline and menopause was 58.6 and 48.2 years, respectively, which means that an average of 10 years have passed and the recall bias of age at natural menopause is inevitable.

## Conclusions

In summary, our large prospective study provided convincing evidence that women had significantly higher risk of developing diabetes during the menopausal transition and post-menopause stages. In particular, among post-menopausal women, we found that both earlier and later age at natural menopause were associated with increased risk of diabetes. These findings underlined the significance of the proposed “menopausal transition window” and “estrogen window” where early monitoring and intervention strategies should be introduced in women’s midlife to reduce diabetes risk.

## Supplementary Information

**Additional file 1.** **Table S1:** Adjusted hazard ratios (95% CIs) of diabetes by age at natural menopause within subgroups among post-menopausal women only. **Table S2:** Sensitivity analyses: Adjusted hazard ratios (95% CIs) of diabetes by the menopausal status and age at natural menopause.

## Data Availability

The data sets generated and/or analysed during the current study are not publicly available due individual privacy information protection but are available from the corresponding author on reasonable request.

## Abbreviations

HR: Hazard ratio
CI: Confidence intervals
IDF: International Diabetes Federation
CKB: China Kadoorie Biobank
OC: Oral contraceptive
RPG: Random plasma glucose
BMI: Body mass index
OR: Odds ratio
