# Supplementary material for: Menopausal status, age at natural menopause and risk of diabetes in China: a 10-year prospective study of 300,000 women
Source: Nutr Metab (Lond). 2022 Feb 5;19:7. doi: 10.1186/s12986-022-00643-x (PMC8818141; doi:10.1186/s12986-022-00643-x)
Supplement: Supplementary file 1 — Additional file 1. Table S1: Adjusted hazard ratios (95% CIs) of diabetes by age at natural menopause within subgroups among post-menopausal women only. Table S2: Sensitivity analyses: Adjusted hazard ratios (95% CIs) of diabetes by the menopausal status and age at natural menopause. [file 12986_2022_643_MOESM1_ESM.docx]

| Table S1. Adjusted hazard ratios (95% CIs) of diabetes by age at natural menopause within subgroups among post-menopausal women only. | | | | | | |
| --- | --- | --- | --- | --- | --- | --- |
|  | Total/cases | <40 | 40-44 | 45-49 | 50-53 | ≧54 |
| Region |  |  |  |  |  |  |
| Rural | 77,720/4,520 | 1.13 (0.95-1.33) | 1.04 (0.94-1.15) | 1.00 | 1.15 (1.07-1.24) * | 1.08 (0.96-1.22) |
| Urban | 64,069/3,220 | 1.17 (0.96-1.42) | 0.96 (0.83-1.10) | 1.00 | 1.03 (0.95-1.12) | 1.10 (0.97-1.25) |
| Heterogeneity test: χ^2^ (P) |  | 0.07 (0.79) | 0.82 (0.37) | NA | 3.82 (0.05) | 0.04 (0.84) |
| Birth cohorts |  |  |  |  |  |  |
| 1920s-1930s | 26,569/1,582 | 1.16 (0.89-1.48) | 0.94 (0.78-1.12) | 1.00 | 1.15 (1.02-1.30) * | 1.09 (0.90-1.30) |
| 1940s | 54,450/3,361 | 1.01 (0.80-1.26) | 1.03 (0.90-1.17) | 1.00 | 1.07 (0.99-1.17) | 1.09 (0.97-1.21) |
| 1950s-1970s | 60,770/2,797 | 1.26 (1.03-1.53) * | 1.05 (0.92-1.19) | 1.00 | 1.11 (1.01-1.21) * | 1.13 (0.90-1.40) |
| Heterogeneity test: χ^2^ (P) |  | 2.08 (0.35) | 1.00 (0.61) | NA | 0.98 (0.61) | 0.09 (0.96) |
| Education |  |  |  |  |  |  |
| No formal school | 51,978/3,182 | 0.99 (0.81-1.20) | 1.04 (0.92-1.18) | 1.00 | 1.11 (1.02-1.20) * | 1.06 (0.93-1.22) |
| Primary school | 50,845/2,848 | 1.35 (1.08-1.66) * | 1.04 (0.90-1.19) | 1.00 | 1.13 (1.03-1.23) * | 1.17 (1.02-1.35) * |
| Middle school or higher | 38,966/1,710 | 1.20 (0.90-1.56) | 0.90 (0.75-1.09) | 1.00 | 1.04 (0.93-1.17) | 1.05 (0.87-1.27) |
| Heterogeneity test: χ^2^ (P) |  | 4.46 (0.11) | 1.84 (0.40) | NA | 1.32 (0.52) | 1.26 (0.53) |
| Regular smoking |  |  |  |  |  |  |
| No | 13,6650/7,394 | 1.15 (1.01-1.31) * | 1.00 (0.92-1.09) | 1.00 | 1.09 (1.03-1.15) * | 1.11 (1.02-1.21) * |
| Yes | 5,139/346 | 1.15 (0.61-1.97) | 1.12 (0.79-1.57) | 1.00 | 1.34 (1.04-1.73) * | 0.75 (0.44-1.21) |
| Heterogeneity test: χ^2^ (P) |  | 0.00 (1.00) | 0.39 (0.53) | NA | 2.42 (0.12) | 2.24 (0.13) |
| Regular alcohol drinking |  |  |  |  |  |  |
| No | 136,737/7,470 | 1.15 (1.01-1.31) * | 1.02 (0.94-1.11) | 1.00 | 1.11 (1.05-1.17) * | 1.10 (1.01-1.20) * |
| Yes | 5,052/270 | 0.95 (0.46-1.75) | 0.74 (0.46-1.15) | 1.00 | 0.94 (0.70-1.26) | 1.10 (0.67-1.73) |
| Heterogeneity test: χ^2^ (P) |  | 0.30 (0.58) | 1.82 (0.18) | NA | 1.19 (0.28) | 0.00 (1.00) |
| Body mass index, ㎏/㎡ |  |  |  |  |  |  |
| <18.5 | 7,849/199 | 2.00 (0.99-3.68) | 1.57 (1.03-2.36) * | 1.00 | 1.25 (0.87-1.80) | 1.46 (0.80-2.52) |
| 18.5-23.9 | 68,000/2,399 | 1.20 (0.96-1.50) | 1.06 (0.92-1.22) | 1.00 | 1.15 (1.05-1.27) * | 1.04 (0.87-1.23) |
| 24.0-27.9 | 48,054/3,271 | 1.08 (0.87-1.32) | 1.04 (0.92-1.18) | 1.00 | 1.12 (1.03-1.22) * | 1.13 (0.99-1.28) |
| ≧28.0 | 17,885/1,871 | 1.08 (0.83-1.38) | 0.81 (0.67-0.97) * | 1.00 | 0.99 (0.88-1.10) | 1.08 (0.92-1.27) |
| Heterogeneity test: χ^2^ (P) |  | 3.45 (0.33) | 10.47 (0.02) * | NA | 4.91 (0.18) | 1.56 (0.67) |
| Hypertension |  |  |  |  |  |  |
| No | 80,261/3,200 | 1.17 (0.95-1.41) | 1.00 (0.88-1.13) | 1.00 | 1.12 (1.03-1.22) * | 1.12 (0.97-1.29) |
| Yes | 61,528/4,540 | 1.13 (0.95-1.33) | 1.02 (0.91-1.13) | 1.00 | 1.09 (1.01-1.17) * | 1.09 (0.97-1.21) |
| Heterogeneity test: χ^2^ (P) |  | 0.07 (0.79) | 0.06 (0.81) | NA | 0.23 (0.64) | 0.09 (0.77) |
| Age at menarche, year |  |  |  |  |  |  |
| ≦12 | 5,235/272 | 0.65 (0.31-1.20) | 0.73 (0.48-1.06) | 1.00 | 0.97 (0.72-1.31) | 0.84 (0.47-1.40) |
| 13-14 | 29,079/1,612 | 1.06 (0.80-1.37) | 0.92 (0.78-1.09) | 1.00 | 0.97 (0.86-1.10) | 1.14 (0.93-1.38) |
| 15-16 | 51,957/2,935 | 1.28 (1.03-1.57) * | 1.09 (0.95-1.24) | 1.00 | 1.13 (1.03-1.23) * | 1.20 (1.04-1.38) * |
| 17-18 | 42,718/2,306 | 1.24 (0.97-1.56) | 1.07 (0.92-1.25) | 1.00 | 1.16 (1.05-1.28) * | 1.04 (0.89-1.22) |
| ≧19 | 12,800/615 | 0.90 (0.53-1.43) | 0.85 (0.61-1.16) | 1.00 | 1.16 (0.96-1.40) | 1.04 (0.78-1.37) |
| Heterogeneity test: χ^2^ (P) |  | 5.39 (0.25) | 6.68 (0.15) | NA | 6.45 (0.17) | 3.10 (0.54) |
| Oral contraceptive use |  |  |  |  |  |  |
| Never | 127,830/6,883 | 1.09 (0.95-1.24) | 1.01 (0.93-1.10) | 1.00 | 1.11 (1.05-1.17) * | 1.08 (0.99-1.19) |
| Ever | 13,959/857 | 1.89 (1.29-2.68) * | 0.97 (0.75-1.26) | 1.00 | 1.05 (0.89-1.24) | 1.26 (0.97-1.62) |
| Heterogeneity test: χ^2^ (P) |  | 7.69 (0.01) * | 0.08 (0.77) | NA | 0.39 (0.53) | 1.23 (0.27) |
| Number of live births † |  |  |  |  |  |  |
| One | 24,613/1,137 | 1.35 (1.02-1.78) * | 1.06 (0.87-1.29) | 1.00 | 1.14 (0.99-1.33) | 1.14 (0.79-1.61) |
| Two | 43,329/2,396 | 1.09 (0.85-1.39) | 1.04 (0.89-1.20) | 1.00 | 1.03 (0.94-1.14) | 1.02 (0.87-1.20) |
| Three | 35,470/2,009 | 1.02 (0.75-1.34) | 1.08 (0.91-1.26) | 1.00 | 1.09 (0.99-1.21) | 1.04 (0.89-1.22) |
| Four or more | 36,579/2,107 | 1.20 (0.94-1.50) | 0.93 (0.79-1.08) | 1.00 | 1.16 (1.04-1.28) * | 1.20 (1.03-1.39) * |
| Heterogeneity test: χ^2^ (P) |  | 2.22 (0.53) | 2.02 (0.57) | NA | 3.03 (0.39) | 2.64 (0.45) |
| Age at first birth, year † |  |  |  |  |  |  |
| <20 | 20,956/1,560 | 1.11 (0.82-1.46) | 1.13 (0.94-1.34) | 1.00 | 1.21 (1.08-1.37) * | 1.09 (0.89-1.31) |
| 20-24 | 74,341/4,125 | 1.15 (0.97-1.36) | 0.96 (0.86-1.08) | 1.00 | 1.07 (0.99-1.15) | 1.12 (1.00-1.26) * |
| 25-29 | 39,434/1,746 | 1.18 (0.88-1.55) | 1.02 (0.85-1.21) | 1.00 | 1.14 (1.02-1.27) * | 1.09 (0.89-1.32) |
| ≧30 | 5,260/218 | 1.26 (0.55-2.52) | 0.99 (0.59-1.57) | 1.00 | 0.78 (0.56-1.08) | 0.79 (0.44-1.33) |
| Heterogeneity test: χ^2^ (P) |  | 0.14 (0.99) | 2.32 (0.51) | NA | 7.58 (0.06) | 1.49 (0.69) |
| Breastfeeding per child, month † |  |  |  |  |  |  |
| ≦6 | 11,068/622 | 1.64 (1.13-2.32) * | 1.13 (0.85-1.48) | 1.00 | 1.32 (1.08-1.60) * | 1.12 (0.79-1.56) |
| 7-12 | 65,900/3,825 | 1.03 (0.85-1.25) | 0.95 (0.84-1.06) | 1.00 | 1.09 (1.01-1.18) * | 1.11 (0.98-1.25) |
| 13-18 | 30,726/1,694 | 1.14 (0.86-1.49) | 1.02 (0.86-1.21) | 1.00 | 1.04 (0.93-1.17) | 1.01 (0.83-1.20) |
| 19-24 | 20,854/1,008 | 1.19 (0.83-1.65) | 1.09 (0.87-1.35) | 1.00 | 1.12 (0.96-1.30) | 1.21 (0.96-1.52) |
| ≧25 | 11,443/500 | 1.13 (0.66-1.79) | 1.15 (0.85-1.54) | 1.00 | 1.15 (0.93-1.43) | 1.06 (0.74-1.48) |
| Heterogeneity test: χ^2^ (P) |  | 5.04 (0.28) | 2.90 (0.58) | NA | 4.49 (0.34) | 1.56 (0.82) |
| All models were stratified by age and study area and adjusted for education, household income, smoking, alcohol drinking, physical activity (Metabolic Equivalents of Task, h/d), body mass index, waist circumference, hypertension, family history of diabetes, age at menarche, number of live births, age at first birth, breastfeeding duration per child, and oral contraceptive use.  * Significant results.  † Among parous women only. | | | | | | |

| Table S2. Sensitivity analyses: Adjusted hazard ratios (95% CIs) of diabetes by the menopausal status and age at natural menopause. | | | | | | |
| --- | --- | --- | --- | --- | --- | --- |
|  | Total/cases | Model 1 | | Model 2 | Model 3 | Model 4 |
| Excluding women with smoking, alcohol drinking and use of oral contraceptive | | | | | | |
| Menopausal status |  | |  |  |  |  |
| Pre- | 65,749/1,607 | | 1.00 | 1.00 | 1.00 | 1.00 |
| Peri- | 6,816/355 | | 1.25 (1.09-1.42) * | 1.24 (1.09-1.41) * | 1.22 (1.07-1.39) * | 1.21 (1.06-1.39) * |
| Post- | 82,386/4,758 | | 1.13 (1.01-1.26) * | 1.19 (1.07-1.32) * | 1.17 (1.15-1.31) * | 1.18 (1.05-1.31) * |
| Age at menopause, year |  | |  |  |  |  |
| <40 | 3,396/174 | | 1.02 (0.87-1.20) | 1.03 (0.87-1.20) | 0.99 (0.83-1.17) | 0.98 (0.82-1.16) |
| 40-44 | 9,608/504 | | 1.00 (0.90-1.10) | 1.02 (0.92-1.12) | 1.02 (0.92-1.13) | 1.02 (0.92-1.14) |
| 45-49 | 33,914/1,853 | | 1.00 | 1.00 | 1.00 | 1.00 |
| 50-53 | 28,947/1,789 | | 1.12 (1.04-1.19) * | 1.08 (1.02-1.16) * | 1.08 (1.01-1.16) * | 1.18 (1.01-1.16) * |
| ≧54 | 6,521/438 | | 1.14 (1.02-1.27) * | 1.06 (0.96-1.18) | 1.05 (0.94-1.17) | 1.06 (0.95-1.18) |
| Excluding women with baseline age of <57 years ‡ | | | | | | |
| Menopausal status |  | |  |  |  |  |
| Pre- | 125,357/3,049 | | 1.00 | 1.00 | 1.00 | 1.00 |
| Peri- | 13,943/658 | | 1.18 (1.17-1.29) * | 1.18 (1.07-1.29) * | 1.17 (1.06-1.29) * | 1.16 (1.05-1.28) * |
| Post- | 63,930/3,065 | | 1.11 (1.02-1.20) * | 1.16 (1.07-1.25) * | 1.16 (1.07-1.26) * | 1.15 (1.06-1.25) * |
| Age at menopause, year |  | |  |  |  |  |
| <40 | 3,146/151 | | 1.30 (1.08-1.57) * | 1.27 (1.05-1.53) * | 1.32 (1.09-1.59) * | 1.29 (1.06-1.55) * |
| 40-44 | 8,018/345 | | 1.03 (0.90-1.16) | 1.02 (0.90-1.16) | 1.03 (0.90-1.16) | 1.02 (0.89-1.15) * |
| 45-49 | 28,526/1,268 | | 1.00 | 1.00 | 1.00 | 1.00 |
| 50-53 | 22,233/1,170 | | 1.16 (1.07-1.27) * | 1.12 (1.03-1.22) * | 1.11 (1.02-1.21) * | 1.11 (1.01-1.20) * |
| ≧54 | 2,007/131 | | 1.34 (1.11-1.61) * | 1.21 (1.00-1.45) * | 1.20 (0.99-1.45) | 1.21 (1.00-1.46) * |
| Model 1 adjusted for education, household income; model 2 adjusted for model 1 plus health behaviors of physical activity (Metabolic Equivalents of Task, h/d), and anthropometric measurements including body mass index, waist circumference; model 3 adjusted for model 2 plus health status of hypertension, and family history of diabetes; model 4 adjusted for model 3 plus reproductive factors of age at menarche, number of live births, age at first birth, and breastfeeding duration per child.  All models were stratified by age and study area.  * Significant results.  ‡ Additionally adjusted for smoking, alcohol drinking and oral contraceptive use. | | | | | | |
